# Incidental Gallbladder Cancer After Cholecystectomy for Presumed Benign Biliary Disease: A Sixteen-Year Retrospective Cohort Study from a Tertiary Referral Center

**DOI:** 10.3390/medicina62050915

**Published:** 2026-05-08

**Authors:** Gökay Çetinkaya, Ahmet Başkent, Mehmet Furkan Başkent, Hasan Fehmi Küçük

**Affiliations:** Kartal Dr. Lütfi Kırdar City Hospital, 34862 İstanbul, Türkiye; abaskent@gmail.com (A.B.); furkanbaskent99@gmail.com (M.F.B.); hfkucuk@gmail.com (H.F.K.)

**Keywords:** incidental gallbladder cancer, cholecystectomy, pathological T stage, overall survival, disease-free survival, re-resection, adjuvant therapy

## Abstract

*Background and Objectives*: Incidental gallbladder cancer (IGBC) is an uncommon but clinically important diagnosis after cholecystectomy for presumed benign biliary disease. This study aimed to determine the incidence of invasive IGBC in a large cholecystectomy cohort and to describe its clinicopathological profile, stage-specific management pathway, and exploratory univariable survival associations. *Materials and Methods*: We retrospectively reviewed all cholecystectomies performed between January 2010 and December 2025 at a tertiary referral center (*n* = 19,798). Patients with known preoperative gallbladder cancer and those with incomplete data precluding reliable staging or survival assessment were excluded. Only invasive IGBC was analyzed; dysplasia and carcinoma in situ were excluded a priori. Overall survival (OS) was defined from index surgery to death from any cause, and disease-free survival (DFS) was assessed in patients with non-metastatic disease at baseline. Survival was estimated using Kaplan–Meier methods, and associations with survival outcomes were explored using univariable Cox regression. *Results*: IGBC was identified in 43 patients (0.22%). Adenocarcinoma predominated, pT2 was the most frequent pathological stage, and 11 patients (25.6%) had pT3–T4 disease. Staged re-resection was performed in 12 patients (27.9%). Median OS was 48.0 months (95% CI, 34.0–62.0), and median DFS in the M0 cohort was 80.0 months (95% CI, 9.5–150.5). The 2-, 4-, and 6-year OS rates were 78.2%, 48.3%, and 38.8%, respectively; the corresponding DFS rates were 70.4%, 59.3%, and 50.8%. In exploratory univariable analyses, pathological T stage showed the most consistent unadjusted association with OS and DFS, whereas margin positivity, perineural invasion, lymphovascular invasion, and increasing tumor size were associated with worse DFS. *Conclusions*: Although rare, IGBC may present with advanced pathological features despite presumed benign disease. These findings support meticulous pathological assessment, structured postoperative staging, and risk-adapted multidisciplinary management. Given the limited sample size and exploratory, unadjusted analyses, survival associations should be interpreted cautiously.

## 1. Introduction

Gallbladder cancer (GBC) is an aggressive malignancy characterized by rapid progression, poor prognosis, and high mortality, and it remains difficult to treat effectively [1]. It accounts for approximately 4% of all gastrointestinal cancers and represents the most common malignancy of the biliary tract [2,3]. Most cases are highly aggressive adenocarcinomas and contribute to a considerable disease burden, including approximately 3710 deaths annually in the United States [2,4,5]. Even with treatment, outcomes remain poor, with reported median overall survival ranging from 3 to 22 months [6,7,8]. The etiology of GBC is multifactorial and includes gallstones, a calcified gallbladder wall, adenomatous polyps, obesity, estrogen exposure, choledochal cysts, and chemical carcinogens [9,10]. Among these factors, gallstones are considered the strongest risk factor and are implicated in 75–90% of cases [9,11].

In patients undergoing cholecystectomy for presumed benign indications without preoperative suspicion of malignancy, the detection of carcinoma on histopathological examination of the cholecystectomy specimen is defined as incidental gallbladder cancer (IGBC) [1,2,3,12]. The reported incidence of IGBC ranges from 0.25% to 3.3% [13], and approximately 27–41% of all GBCs are diagnosed incidentally [14]. With the global expansion of laparoscopic cholecystectomy, a corresponding increase in the detection of IGBC has been reported, highlighting the clinical relevance of this entity in contemporary surgical practice [1,15].

The primary reason IGBC remains undetected preoperatively is the lack of specific and reliable clinical or radiological findings. Although several warning signs have been described—such as irregular gallbladder wall thickening, large polyps, nonvisualization of the gallbladder, or lymphadenopathy—these features do not consistently or reliably prompt preoperative suspicion of malignancy in routine clinical practice [2,16,17]. Consequently, a key unresolved issue in IGBC management is the optimal oncologic strategy after an unexpected diagnosis, including whether completion surgery should be performed, the appropriate timing of re-intervention, and the extent of resection required for oncologic adequacy [1,15]. In many patients, a second surgical procedure may be necessary to achieve oncologic completeness, yet the survival benefit of additional resection across GBC stages remains debated [15]. This uncertainty is further amplified in real-world practice by patient-level factors common in IGBC cohorts—particularly advanced age and comorbidity—which may limit eligibility for, or acceptance of, completion surgery despite guideline-based recommendations [1]. Accordingly, IGBC should be viewed not only as an incidental pathological diagnosis but also as a clinically challenging postoperative management problem that frequently requires individualized decision-making.

In this retrospective study, we evaluated the clinical, surgical, and pathological characteristics of patients diagnosed with IGBC among cholecystectomy cases performed at our institution between 2010 and 2025, described the real-world stage-specific management pathway after incidental diagnosis, and compared these findings with the existing literature to inform contemporary IGBC management.

## 2. Materials and Methods

### 2.1. Study Design and Patient Selection

This retrospective cohort study was conducted using prospectively archived surgical and pathology databases from a tertiary referral center. In the ethics committee application file, the period from January 2010 to December 2025 was defined as the clinical archive period to be reviewed retrospectively, and it was stated that research-related record review, data extraction, and analysis activities would begin after December 2025. Patients with a known preoperative diagnosis of gallbladder cancer (GBC) (*n* = 15) and those with incomplete clinical or pathological data precluding reliable staging or survival assessment (*n* = 376) were excluded. The remaining 19,407 patients underwent cholecystectomy for presumed benign biliary disease and constituted the study population.

IGBC was defined as malignancy not suspected preoperatively and diagnosed either intraoperatively or on postoperative histopathological examination of the cholecystectomy specimen. Within this cohort, IGBC was identified in 43 patients (0.22%). Malignancy was recognized intraoperatively in 4 patients and detected on final histopathological examination in 39 patients. DFS analysis was restricted to patients with non-metastatic disease at baseline (M0). All 36 M0 patients were included in the DFS analysis.

**Figure 1:**
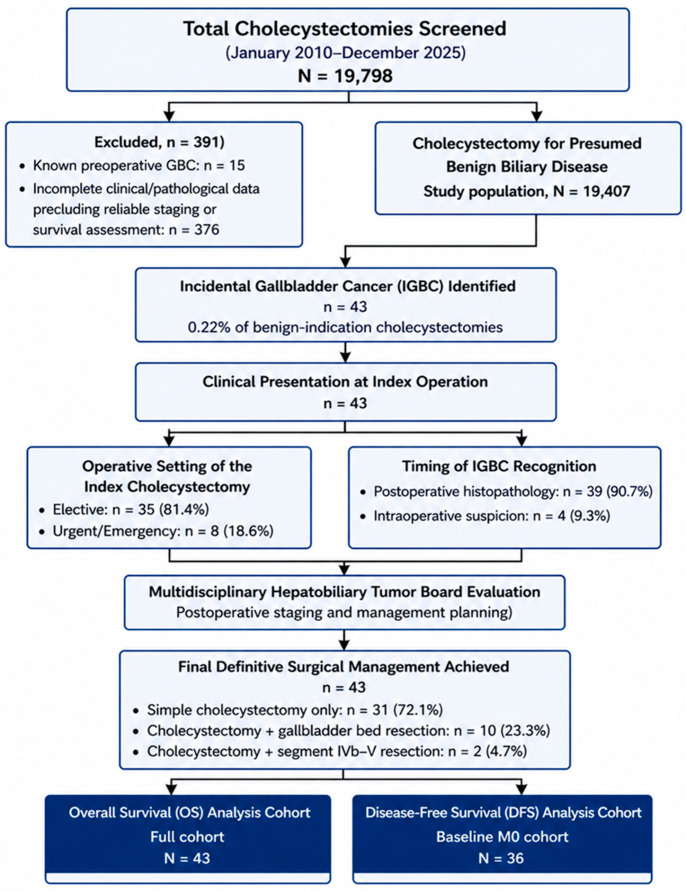
Flowchart of patient selection, diagnostic timing, and staged management for IGBC in a tertiary referral center (January 2010–December 2025).

Only patients with invasive carcinoma were included in the analysis. Dysplasia and carcinoma in situ (Tis) were excluded a priori because these lesions are biologically distinct from invasive disease, lack invasive potential, and would introduce outcome misclassification and artificially inflate survival estimates. This study included invasive IGBC across the spectrum from pT1a to pT4. T1a lesions were retained because they represent invasive carcinoma and therefore belong to the real-world clinicopathological spectrum of IGBC, whereas dysplasia and carcinoma in situ were excluded as non-invasive lesions. The complete selection and management pathway, including diagnosis timing, staging, multidisciplinary evaluation, and definitive treatment, are summarized in Figure 1.

### 2.2. Data Collection, Definitions and Study Variables

Demographic and clinical data were extracted from institutional electronic records. These included age, sex, body mass index (BMI), American Society of Anesthesiologists (ASA) physical status, and comorbidity burden. Comorbidity was quantified using the Charlson Comorbidity Index (CCI), calculated based on documented diagnoses at the time of index admission, and summarized descriptively.

Preoperative clinical history included prior cholecystitis, choledocholithiasis, pancreatitis, smoking, alcohol use, previous abdominal surgery, and endoscopic retrograde cholangiopancreatography (ERCP). Preoperative ultrasonographic findings, including gallbladder wall thickness, primary indication for surgery, and gallstone size, were recorded.

Preoperative laboratory parameters were defined as values obtained before the index cholecystectomy. Tumor markers, including CA 19-9 and carcinoembryonic antigen (CEA), were assessed only after histopathological confirmation as part of the postoperative staging work-up.

Histopathological evaluation included tumor subtype, anatomical location, histologic grade, lymphovascular invasion, perineural invasion, and resection margin status. Margin status was classified as negative (R0), cystic duct positive, liver bed positive, or not assessable. Tumor staging was performed according to the 8th edition of the American Joint Committee on Cancer (AJCC). Distant metastasis (M stage) was determined using postoperative cross-sectional imaging performed after histopathological diagnosis.

We specifically documented intraoperative suspicion of malignancy based on operative reports. Suspicion criteria included a macroscopic mass, irregular or nodular gallbladder wall thickening, induration, suspected hepatic invasion, or abnormal cystic duct margin.

Postoperative complications were classified according to the surgical procedure and timing (index surgery versus reoperation). Because postoperative severity grading could not be reconstructed uniformly and reliably from the retrospective records across the full study period, complications were recorded descriptively and were not retrospectively reclassified using the Clavien–Dindo system. Complications, including bile leak, surgical site infection, and postoperative hemorrhage, were recorded and attributed to the corresponding surgical stage.

### 2.3. Surgical Management Strategy and Criteria for Additional Surgery

All index procedures were planned as standard cholecystectomy for presumed benign disease. Because these operations were not scheduled as oncologic resections, frozen-section analysis was not routinely performed. Even in cases with intraoperative macroscopic suspicion of malignancy, the surgical procedure was not extended during the same session.

This strategy was adopted because major hepatic resection and systematic lymphadenectomy are associated with substantial morbidity and require specific preoperative oncologic consent and staging. Moreover, intraoperative radicalization without confirmed histopathology and systemic staging may result in unnecessary major surgery in patients with occult metastatic disease. Therefore, intraoperative extended resection was intentionally avoided.

After histopathological diagnosis, all patients were evaluated by a multidisciplinary hepatobiliary tumor board. Eligibility for curative-intent staged re-resection was assessed after postoperative staging. Patients were considered eligible for staged re-resection if they had pathological stage ≥T1b, positive cystic duct or liver bed margin, or radiological suspicion of residual disease, provided that distant metastasis was absent and surgical risk was acceptable. Patients with T1a disease without additional high-risk indications, distant metastasis, poor performance status, or prohibitive surgical risk were not considered eligible for staged re-resection. Patient preference was recorded only among patients who were clinically eligible but did not undergo re-resection.

Definitive surgical management included simple cholecystectomy alone (*n* = 31), gallbladder bed resection (*n* = 10), and anatomical segment IVb–V resection (*n* = 2). Hepatic pedicle lymphadenectomy was performed selectively during re-resection. Lymph node yield and nodal involvement were recorded.

Margin extension procedures, including cystic duct margin re-excision and bile duct resection, were documented when performed. Re-resection status could be reliably ascertained from operative and pathological records; however, exact date-level information required to calculate the interval from index cholecystectomy to oncologic re-resection was not available in a complete and uniform manner for all 12 re-resected patients. In several cases, the records documented that re-resection had been performed but did not consistently preserve all date fields needed for reproducible interval calculation, including the date of referral, completion of postoperative staging, multidisciplinary tumor board decision, and final operative scheduling. Because calculating a median interval using only the subset with complete date-level information would have introduced selective reporting and potential information bias, the interval to re-resection was not analyzed quantitatively.

Adjuvant therapy eligibility was determined based on tumor stage, margin status, nodal involvement, and multidisciplinary tumor board recommendations. Data on chemotherapy regimens, chemoradiotherapy, and reasons for omission (such as early-stage disease, comorbidity, postoperative complications, or patient refusal) were collected.

### 2.4. Statistical Analysis

All statistical analyses were performed using IBM SPSS Statistics version 27.0 (IBM Corp., Armonk, NY, USA). Continuous variables were evaluated for normality using histograms, Q–Q plots, and the Shapiro–Wilk test. Normally distributed data were summarized as mean ± standard deviation, whereas non-normally distributed variables were expressed as median and interquartile range. Categorical variables were presented as counts and percentages.

Overall survival (OS) was defined as the interval from index surgery to death from any cause. Disease-free survival (DFS) was analyzed in patients with non-metastatic disease at baseline (M0) and was defined as the interval from index surgery to recurrence or death, whichever occurred first. Because cause-specific mortality adjudication was not consistently available across retrospective follow-up, DFS was interpreted as a composite endpoint rather than a purely recurrence-specific endpoint. Patients without events were censored at last follow-up. Kaplan–Meier curves were generated separately for OS in the full cohort and DFS in the M0 cohort. Censoring marks and number-at-risk tables were displayed below each curve. Median follow-up was estimated using the reverse Kaplan–Meier method.

Univariable Cox proportional hazards regression models were used to explore unadjusted associations between candidate clinicopathological variables and survival outcomes. Given the rarity of IGBC and the limited number of outcome events, survival analyses were treated as exploratory. A multivariable Cox model was not fitted because the number of events was insufficient relative to the number of clinically relevant covariates, including age, comorbidity burden, ASA class, pathological stage, and margin status. Under these conditions, adjusted estimates would be vulnerable to overfitting, unstable confidence intervals, and potentially misleading inference. Hazard ratios with 95% confidence intervals and Wald *p* values were reported. The proportional hazards assumption was assessed using log-minus-log plots and time-dependent covariate tests. Complete-case analysis was performed due to minimal missing data. Accordingly, all reported hazard ratios should be interpreted as exploratory unadjusted associations rather than definitive independent effects, and wide confidence intervals were considered indicators of limited precision rather than definitive effect magnitude. A two-sided p value < 0.05 was considered statistically significant.

### 2.5. Ethical Approval

This study was approved by the Local Ethics Committee of Kartal Dr. Lütfi Kırdar City Hospital (Approval No.: 2025/010.99/21/33; approval date: 30 October 2025). The ethics committee application file specified January 2010–December 2025 as the retrospective clinical archive review period, and this period was included within the approved scope of the ethics committee approval. All procedures involving human participants were conducted in accordance with the ethical standards of the institutional and/or national research committee and the 1964 Declaration of Helsinki and its later amendments or comparable ethical standards.

## 3. Results

A total of 43 patients with invasive IGBC were identified among 19,407 cholecystectomies performed for presumed benign indications. Baseline demographic, clinical, imaging, operative, and diagnostic characteristics are summarized in Table 1. Briefly, the cohort was predominantly elderly and female, with a high comorbidity burden and frequent gallstone disease. Most cases were diagnosed postoperatively on final histopathological examination, whereas intraoperative suspicion of malignancy was uncommon.

Pathological characteristics, staging, treatment pathway, and postoperative outcomes are summarized in Table 2. Adenocarcinoma predominated, pT2 was the most frequent pathological stage, and advanced pathological stage (pT3–T4) was observed in approximately one quarter of patients. Distant metastasis was identified at postoperative staging in 7 patients. After postoperative staging and multidisciplinary review, staged re-resection was performed in 12 patients, whereas 17 clinically eligible patients did not undergo re-resection. Postoperative complications were uncommon and were summarized descriptively because Clavien–Dindo grading could not be uniformly reconstructed.

**Figure 2:**
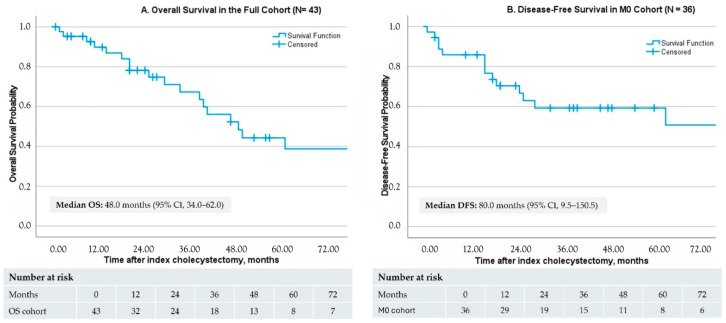
Kaplan–Meier estimates of overall survival and disease-free survival in patients with IGBC. (**A**) Overall survival (OS) in the full cohort (*N* = 43); median OS was 48.0 months (95% CI, 34.0–62.0). (**B**) Disease-free survival (DFS) in the M0 cohort (*N* = 36); median DFS was 80.0 months (95% CI, 9.5–150.5). Number-at-risk tables are shown below each curve. Censoring marks indicate patients without an event at last follow-up. Late time-point estimates should be interpreted cautiously because the number at risk decreased over follow-up. CI, confidence interval; DFS, disease-free survival; IGBC, incidental gallbladder cancer; M0, no distant metastasis; OS, overall survival.

Kaplan–Meier survival estimates are summarized in Table 3 and illustrated in Figure 2. In the full cohort, median OS was 48.0 months (95% CI, 34.0–62.0), with 22 deaths and 21 censored observations. The estimated 2-, 4-, and 6-year OS rates were 78.2%, 48.3%, and 38.8%, respectively. Median follow-up for OS was 55.0 months (95% CI, 43.0–67.0) by reverse Kaplan–Meier estimation. DFS was analyzed in 36 patients with M0 disease at baseline. Median DFS was 80.0 months (95% CI, 9.5–150.5), with 15 DFS events and 21 censored observations. The estimated 2-, 4-, and 6-year DFS rates were 70.4%, 59.3%, and 50.8%, respectively. Median follow-up for DFS was 48.0 months (95% CI, 34.9–61.1). Late estimates should be interpreted cautiously because the number at risk decreased over follow-up.

Exploratory univariable Cox regression analyses for OS are summarized in Table 4. Pathological T stage showed an overall univariable association with OS (overall *p* = 0.038). Urgent surgery was associated with a higher hazard of death (HR 3.47, 95% CI, 1.08–11.15; *p* = 0.037). The univariable association between any comorbidity and lower mortality (HR 0.19, 95% CI, 0.05–0.71; *p* = 0.013) was counterintuitive and should be regarded as clinically non-confirmatory, given the very small comparator group without comorbidity and the potential for sparse-data bias, imbalance, selection bias, and treatment-selection effects. Other clinical, pathological, and laboratory variables were not significantly associated with OS in univariable models. These findings are exploratory and unadjusted and should not be interpreted as independent prognostic effects.

Exploratory univariable Cox regression analyses for DFS in the M0 cohort are summarized in Table 5. Pathological T stage showed a univariable association with worse DFS. Compared with T1 disease, both T2 and T3–T4 tumors were associated with higher hazards of a DFS event, defined as recurrence or death. Resection margin positivity, lymphovascular invasion, perineural invasion, and increasing tumor size were also associated with higher hazards of a DFS event, defined as recurrence or death, in univariable analyses. These findings are exploratory and unadjusted and should not be interpreted as independent prognostic effects.

## 4. Discussion

The present study shows that invasive IGBC, although rare, remains clinically relevant because incidental detection does not exclude advanced pathological stage, adverse histological features, or clinically meaningful survival outcomes. The key clinical message is that IGBC should not be regarded as a biologically indolent finding after presumed benign biliary surgery; rather, it should prompt structured postoperative staging, careful pathological risk assessment, and multidisciplinary review.

The identification of IGBC within a large cholecystectomy population confirms that this entity continues to represent a clinically important problem in routine surgical practice [18,19]. Reported IGBC incidence varies geographically and has been described between 0.25% and 3.3% [13]. Moreover, approximately 27–41% of GBCs are diagnosed incidentally, and residual disease has been reported in a substantial proportion of patients undergoing re-exploration [14]. These findings support the concept that incidental diagnosis does not necessarily imply oncologically adequate treatment at the index operation. Nevertheless, the present results should be interpreted in the context of a tertiary referral center, where case-mix and referral patterns may differ from non-referral settings.

The demographic and clinical profile of this cohort was broadly consistent with previous IGBC series. Advanced age and female sex have repeatedly been described as common risk factors for IGBC [20]. The higher frequency of GBC in women has been linked, at least partly, to estrogen-related changes in bile composition and gallstone formation [21]. Our findings are consistent with Chaturvedi et al., who also reported a female predominance [22], whereas Matsuyama et al. observed a lower female proportion, suggesting potential geographic and population-level variation [23]. Age has also been identified as an important predictor in previous studies and systematic reviews [24,25]. Similarly, Ahn et al. reported age ≥65 years as the only independently associated factor in their multivariable model [26]. Taken together, these observations emphasize that IGBC often occurs in older patients whose comorbidity burden and operative risk may affect the feasibility of completion oncologic management.

Gallstone disease remains central to IGBC epidemiology. Previous studies have shown that gallstone disease is the most common indication for cholecystectomy among patients ultimately diagnosed with IGBC [27]. However, reliable clinical or radiological features that consistently predict early GBC or premalignant lesions in patients with gallstones remain lacking [28]. Gallstones are widely recognized as the strongest risk factor for GBC and have been implicated in 75–90% of cases [9,11]. The high frequency of gallstone disease in the present cohort is therefore consistent with the established literature and reinforces that malignancy may remain occult despite a presumed benign indication for cholecystectomy.

The pathological profile of the cohort further supports the need for careful postoperative histopathological assessment. Adenocarcinoma is the predominant histological subtype of GBC, accounting for approximately 90–95% of malignant gallbladder neoplasms [21]. Most IGBC cases not suspected preoperatively are reported at pT1–pT2 stages [14,29], and pT2 was also the most frequent stage in the present cohort. However, the presence of pT3–T4 disease in a clinically relevant subset indicates that incidental detection does not preclude advanced tumor biology. This pattern is comparable to previous series, including Chaturvedi et al. [22] and Wu et al. [3]. In addition, the observed rates of perineural and lymphovascular invasion were in line with those reported by Ethun et al. [30], underscoring that adverse pathological features should not be overlooked in IGBC.

Surgical management after incidental diagnosis remains complex. For GBC, surgical resection is the only potentially curative treatment, and radical cholecystectomy is generally regarded as the standard oncologic approach [14]. Radical cholecystectomy includes removal of the gallbladder with hepatic bed resection involving segments IV and V and regional lymph node dissection [14]. Current guidance supports re-resection after laparoscopic cholecystectomy in appropriately selected patients with T1b, T2, or T3 IGBC [31]. Consistent with this framework, staged re-resection in the present cohort was considered only after postoperative confirmation, staging, and multidisciplinary review. These findings support a staged, risk-adapted approach in which patients with ≥T1b disease, positive margins, suspected residual disease, or other adverse pathological features are evaluated for completion oncologic surgery after postoperative staging and multidisciplinary review. However, the final treatment decision should also account for metastatic status, operative fitness, comorbidity burden, competing clinical risks, and patient preference. Similar barriers to reoperation have been reported by Wu et al. [3], who noted limited acceptance of radical re-resection despite recommendations and identified advanced age as a major reason for refusal [3]. Likewise, Ahn et al. reported that many patients with pT2–pT3 disease were managed with cholecystectomy alone because of advanced age and comorbidity [26]. Because re-resection interval data were incomplete, the effect of reoperation timing could not be assessed.

Pathological T stage is widely regarded as one of the most important prognostic determinants in GBC because it reflects tumor burden and biological aggressiveness [32,33]. Wu et al. reported that advanced T stage was significant in univariable analyses of poor prognosis [3], and Mazer et al. demonstrated that T stage was a significant predictor of mortality [34]. In the present cohort, pathological T stage showed the most consistent univariable association with both OS and DFS; however, this finding should be interpreted as exploratory and unadjusted rather than as evidence of an independent prognostic effect. In exploratory univariable analyses of the M0 cohort, advanced pathological T stage, perineural invasion, lymphovascular invasion, positive surgical margins, and increasing tumor size were associated with worse DFS. Because DFS was defined as recurrence or death, these associations should be interpreted as higher hazards of a composite DFS event rather than as recurrence-specific effects alone. The relevance of perineural and lymphovascular invasion has also been emphasized by Altiok et al. [20]. Similarly, Vega et al. reported worse OS among patients with positive cystic duct margins at index cholecystectomy [35]. In the present cohort, margin positivity was associated with worse DFS but not with a statistically significant difference in OS, suggesting that its effect on overall mortality may be less clear in elderly and clinically heterogeneous cohorts.

When tumor size was analyzed as a continuous variable in univariable analysis, each 10 mm increase was associated with a higher hazard of a composite DFS event, defined as recurrence or death, supporting a hypothesis-generating relationship between tumor burden and DFS. In contrast, Ahn et al. reported that tumor size itself was not significantly associated with clinical outcomes, despite the prognostic relevance of advanced pT stage [26]. The magnitude of several hazard ratios in the present study should therefore be interpreted cautiously because of limited event numbers and wide confidence intervals. The apparent association between any comorbidity and lower mortality should also not be interpreted as a protective clinical effect, because the comparator group without comorbidity was very small and the estimate may be influenced by sparse-data bias, imbalance, selection bias, treatment-selection effects, and competing clinical risks. As emphasized by Korkmaz et al., the rarity of IGBC limits case numbers, reduces statistical power, and increases the risk of type II error [36]. More broadly, the survival patterns observed here should be interpreted within a real-world framework shaped by tumor biology, surgical eligibility, treatment selection, comorbidity burden, and patient-level decision-making.

From a clinical perspective, these findings support three practical implications. First, incidental diagnosis should trigger meticulous pathological review rather than reassurance based on presumed benign presentation. Second, structured postoperative staging and multidisciplinary review remain essential, particularly for patients with ≥T1b disease, positive margins, or adverse histological features. Third, risk-adapted decision-making is especially important in elderly or comorbid patients, in whom guideline-based re-resection may be clinically appropriate but not always feasible or acceptable. These considerations may help guide future studies designed to better distinguish tumor biology from treatment selection effects and to clarify which patient subgroups derive the greatest benefit from additional surgery or adjuvant therapy.

### Limitations

This study has several limitations that should be considered when interpreting its findings. First, the retrospective, single-center design is inherently subject to selection bias, information bias, and residual confounding. Because this was a tertiary referral cohort, the study population may have included older, more complex, or selectively referred patients, which limits direct generalizability to lower-volume or non-referral settings. Second, although the screening denominator was large, IGBC was rare, and the modest number of included cases together with the limited number of outcome events restricted statistical power and the precision of effect estimates, as reflected by wide confidence intervals for several prognostic associations. Accordingly, the survival analyses should be interpreted as exploratory rather than definitive. Third, a multivariable Cox model was not fitted because the number of events was insufficient relative to the number of clinically relevant covariates, and any adjusted model under these conditions would have been vulnerable to overfitting, unstable confidence intervals, and potentially misleading inference; therefore, the reported hazard ratios should not be interpreted as definitive independent effects. Fourth, treatment allocation was heterogeneous and not randomized. Acceptance of re-resection and adjuvant therapy was influenced by age, comorbidity burden, surgical risk, and patient preference, introducing the possibility of confounding by indication and complicating the separation of tumor biology from treatment selection effects. Fifth, although the interval between index cholecystectomy and staged re-resection is clinically relevant, complete date-level information allowing for reproducible calculation of this interval was not available for all re-resected patients. Therefore, reporting a median interval based on an incomplete subset was avoided to prevent selective reporting and potential information bias. Consequently, this study cannot evaluate whether earlier versus later re-resection influenced oncologic outcomes. Sixth, postoperative complications were recorded descriptively, but complication severity could not be retrospectively standardized using the Clavien–Dindo classification system across the full study period. Seventh, because cause-specific mortality adjudication was not consistently available, disease-free survival was necessarily analyzed as a composite endpoint of recurrence or death rather than as a purely recurrence-specific outcome. Finally, the 16-year inclusion period may also have introduced temporal heterogeneity related to changes in laparoscopic practice, staging work-up, and postoperative oncologic management over time. Despite these limitations, the study provides a clinically relevant real-world description of the clinicopathological spectrum, staged management pathway, and exploratory survival associations of IGBC within a large cholecystectomy population.

## 5. Conclusions

Although rare, IGBC remains clinically meaningful and may present with advanced pathological features despite presumed benign disease. In exploratory univariable analyses, pathological T stage showed the most consistent unadjusted association with OS and DFS, while margin positivity, perineural invasion, lymphovascular invasion, and increasing tumor size were associated with worse DFS, reflecting higher hazards of a composite DFS event rather than recurrence-specific effects alone. These findings indicate that incidental diagnosis should not lessen oncologic vigilance and support meticulous pathological assessment, structured postoperative staging, and multidisciplinary evaluation after index cholecystectomy. However, the observed survival associations should be interpreted cautiously given the limited sample size, wide confidence intervals, treatment heterogeneity, and the exploratory, unadjusted nature of the analyses. Within these constraints, the overall pattern of findings supports risk-adapted postoperative management in IGBC, in which completion oncologic surgery should be considered for appropriate candidates after staging and multidisciplinary review, while metastatic status, operative risk, comorbidity burden, competing clinical risks, and patient preference should guide final treatment decisions.

## Figures and Tables

**Table 1:** Baseline demographics, comorbidity, preoperative imaging, and diagnosis timing in IGBC (*n* = 43).

Characteristic	Overall
Age, years, mean ± SD	71.4 ± 9.9
Female sex, *n* (%)	32 (74.4)
BMI category, *n* (%)	Low 7 (16.3); Normal 21 (48.8); High 15 (34.9)
ASA class, *n* (%)	I 1 (2.3); II 20 (46.5); III 15 (34.9); IV 7 (16.3)
Charlson Comorbidity Index, median [min–max]	3 [3–4]
Any comorbidity, *n* (%)	39 (90.7)
Hepatitis history, *n* (%)	1 (2.3)
Cholecystitis history, *n* (%)	24 (55.8)
Choledocholithiasis history, *n* (%)	14 (32.6)
Pancreatitis history, *n* (%)	12 (27.9)
Smoking history, *n* (%)	12 (27.9)
Alcohol use, *n* (%)	5 (11.6)
ERCP history, *n* (%)	9 (20.9)
Gallbladder wall thickness on US, *n* (%)	<3 mm 15 (34.9); 3–10 mm 24 (55.8); >10 mm 4 (9.3)
Primary US finding, *n* (%)	Stone 38 (88.4); Polyp 5 (11.6)
Largest stone size *, *n*/*N* (%)	<1 cm 12/38 (31.6); 1–3 cm 24/38 (63.2); >3 cm 2/38 (5.3)
Operative setting of the index cholecystectomy, *n* (%)	Elective 35 (81.4); Urgent/Emergency 8 (18.6)
Surgical approach, *n* (%)	Laparoscopic 31 (72.1); Open 7 (16.3); Conversion 5 (11.6)
Intraoperative suspicion of malignancy, *n* (%)	4 (9.3)
Postoperative pathology diagnosis, *n* (%)	39 (90.7)
Advanced stage (pT3–T4) in elective setting	8/35 (22.9)
Advanced stage (pT3–T4) in urgent setting	3/8 (37.5)

* Largest stone size is reported among patients with gallstones on ultrasonography (*N* = 38). ASA, American Society of Anesthesiologists physical status; BMI, body mass index; CCI, Charlson Comorbidity Index; ERCP, endoscopic retrograde cholangiopancreatography; SD, standard deviation; US, ultrasonography.

**Table 2:** Pathological characteristics, staging, treatment pathway, and postoperative outcomes in patients with IGBC.

Characteristic	Overall Cohort, *n* = 43
**Pathological and tumor-related characteristics**	
Histological subtype, *n* (%)	Adenocarcinoma 39 (90.7); other 4 (9.3)
Tumor location, *n* (%)	Fundus 3 (7.0); body 26 (60.5); neck 2 (4.7); diffuse 12 (27.9)
Histological grade, *n* (%)	Low 9 (20.9); intermediate 20 (46.5); high 14 (32.6)
Pathological T stage, AJCC 8th edition, *n* (%)	T1a 7 (16.3); T1b 7 (16.3); T2 18 (41.9); T3 8 (18.6); T4 3 (7.0)
Advanced pathological stage, pT3–T4, *n* (%)	11 (25.6)
Distant metastasis at postoperative staging, *n* (%)	M0 36 (83.7); M1 7 (16.3)
Tumor size, mm, median [IQR]	15 [33]
Perineural invasion, *n* (%)	19 (44.2)
Lymphovascular invasion, *n* (%)	17 (39.5)
**Definitive surgical management**	
Final surgical management achieved, *n* (%)	Simple cholecystectomy only 31 (72.1); cholecystectomy + gallbladder bed resection 10 (23.3); cholecystectomy + segment IVb–V resection 2 (4.7)
Same-session intraoperative oncologic extension during index surgery, *n* (%)	0 (0.0)
Type of same-session intraoperative extension, *n*	None
Staged re-resection after postoperative diagnosis and staging, *n* (%)	12 (27.9)
Indication for staged re-resection among reoperated patients, *n*/*N* (%)	≥T1b stage 12/12 (100.0); positive margin 3/12 (25.0); radiological residual disease/risk 0/12 (0.0)
**Re-resection decision pathway after postoperative staging**	
Not eligible for curative-intent staged re-resection because of T1a disease without additional high-risk indication, *n*	7
Not eligible for curative-intent staged re-resection because of distant metastasis, *n*	7
Not eligible because of poor performance status or prohibitive surgical risk, *n*	0
Eligible after postoperative staging and underwent staged re-resection, *n*	12
Eligible after postoperative staging but did not undergo staged re-resection, *n*	17
—Documented patient refusal	16
—Other or incompletely documented reason	1
**Lymphadenectomy and nodal status**	
Hepatic pedicle lymphadenectomy performed, *n* (%)	12 (27.9)
Lymph node yield, median [IQR]	7 [6]
Node-positive disease among reoperated patients, *n*/*N* (%)	3/12 (25.0)
**Final resection margin status**	
Negative margin, R0, *n* (%)	29 (67.4)
Cystic duct margin positive, *n* (%)	5 (11.6)
Liver bed margin positive, *n* (%)	5 (11.6)
Margin not assessable, *n* (%)	4 (9.3)
Cystic duct margin extension required, *n* (%)	5 (11.6)
Bile duct resection required, *n* (%)	0 (0.0)
**Adjuvant treatment**	
Adjuvant therapy administered, *n* (%)	Yes 29 (67.4); no 14 (32.6)
Chemotherapy regimen, *n* (%)	Capecitabine 24 (55.8); gemcitabine–cisplatin 5 (11.6)
Adjuvant chemoradiotherapy, *n* (%)	2 (4.7)
Reason for omission of adjuvant therapy, *n*	Early-stage/low-risk disease 7; poor performance status/comorbidity 3; postoperative complications 1; patient refusal 3; loss to follow-up/other 0
**Postoperative complications**	
Postoperative complications, *n* (%)	None 41 (95.3); bile leak 1 (2.3); superficial surgical site infection 0 (0.0); postoperative hemorrhage 0 (0.0); other 1 (2.3)
Complications by final surgical management, *n*	Simple cholecystectomy only 2; gallbladder bed resection 0; segment IVb–V resection 0

Re-resection eligibility was assessed after postoperative staging and multidisciplinary hepatobiliary tumor board review. Patients with distant metastasis, T1a disease without additional high-risk indications, or prohibitive surgical risk were not classified as eligible for curative-intent staged re-resection. Patient refusal was recorded only among clinically eligible patients who did not undergo re-resection. Postoperative complications were summarized descriptively because Clavien–Dindo severity grading could not be uniformly reconstructed from the retrospective records. The interval from index cholecystectomy to staged re-resection was not reported because reliable and complete interval data could not be reconstructed across all re-resected patients. AJCC, American Joint Committee on Cancer; IQR, interquartile range; M0, no distant metastasis; M1, distant metastasis; R0, margin-negative resection. Bold formatting indicates category and subsection headings within the table.

**Table 3:** Kaplan–Meier-estimated OS and DFS outcomes in IGBC.

Endpoint	Analysis Cohort	N	Events, n (%)	Censored, n (%)	Median Survival, Months (95% CI)	Mean Survival, Months (95% CI)	2-Year Survival, %	4-Year Survival, %	6-Year Survival, %	Median Follow-Up, Months (95% CI)
OS	Full cohort	43	22 (51.2)	21 (48.8)	48.0 (34.0–62.0)	60.7 (44.8–76.6)	78.2	48.3	38.8	55.0 (43.0–67.0)
DFS	M0 cohort	36	15 (41.7)	21 (58.3)	80.0 (9.5–150.5)	79.6 (55.6–103.5)	70.4	59.3	50.8	48.0 (34.9–61.1)

Values were estimated using the Kaplan–Meier method. OS was defined as time from index cholecystectomy to all-cause death; DFS was defined as recurrence or death, whichever occurred first, in the M0 cohort. Median follow-up was estimated using the reverse Kaplan–Meier method. Late survival estimates should be interpreted cautiously when few patients remain at risk. CI, confidence interval; DFS, disease-free survival; M0, no distant metastasis; OS, overall survival.

**Table 4:** Exploratory univariable Cox proportional hazards regression for OS in IGBC.

Predictor (Unit/Contrast; Reference)	HR	95% CI	*p* Value
Pathological T stage (grouped; overall test)	—	—	**0.038**
├─ T2 vs. T1 (T1)	0.38	0.13–1.09	0.071
└─ T3–T4 vs. T1 (T1)	2.26	0.65–7.81	0.200
Resection margin positivity (R1/R2 vs. R0; R0)	1.41	0.49–4.04	0.524
Lymphovascular invasion (present vs. absent; absent)	1.56	0.54–4.49	0.411
Perineural invasion (present vs. absent; absent)	1.14	0.40–3.29	0.807
High histologic grade (high vs. low; low/intermediate)	0.46	0.17–1.23	0.124
Tumor size (per 10 mm increase)	0.91	0.65–1.25	0.551
Age (per 1-year increase)	0.99	0.94–1.04	0.664
Any comorbidity (yes vs. no; no)	0.19	0.05–0.71	**0.013**
CA 19-9 (log-transformed; per 1-unit increase)	0.87	0.65–1.16	0.345
ASA class (III–IV vs. I–II; I–II)	0.53	0.22–1.30	0.167
Hypoalbuminemia (<3.5 vs. ≥3.5 g/dL; ≥3.5)	0.50	0.11–2.21	0.359
Urgent surgery (urgent vs. elective; elective)	3.47	1.08–11.15	**0.037**
Distant metastasis (M1 vs. M0; M0)	1.70	0.39–7.49	0.484

ASA, American Society of Anesthesiologists physical status; CA 19-9, carbohydrate antigen 19-9; CI, confidence interval; HR, hazard ratio; IGBC, incidental gallbladder cancer; OS, overall survival. All HRs are unadjusted and should be interpreted as exploratory associations. Statistically significant results (*p* < 0.05) are presented in bold.

**Table 5:** Exploratory univariable Cox proportional hazards regression for DFS in IGBC, restricted to the M0 cohort.

Predictor (Unit/Contrast; Reference)	HR	95% CI	*p* Value
**Pathological T stage (grouped; overall test)**	—	—	**0.013**
├─ T2 vs. T1 (T1)	9.51	1.19–75.77	**0.033**
└─ T3–T4 vs. T1 (T1)	27.61	2.90–262.44	**0.004**
Resection margin positivity (R1/R2 vs. R0; R0)	5.91	1.81–19.26	**0.003**
Lymphovascular invasion (present vs. absent; absent)	6.37	2.00–20.33	**0.002**
Perineural invasion (present vs. absent; absent)	15.26	3.26–71.34	**0.001**
High histologic grade (high vs. low; low/intermediate)	0.72	0.24–2.18	0.559
Tumor size (per 10 mm increase)	1.92	1.44–2.57	**<0.001**
Age (per 1-year increase)	1.05	0.99–1.12	0.132
Hypoalbuminemia (<3.5 vs. ≥3.5 g/dL; ≥3.5)	2.68	0.81–8.86	0.106

CI, confidence interval; DFS, disease-free survival; HR, hazard ratio; IGBC, incidental gallbladder cancer; M0, no distant metastasis. All HRs are unadjusted and should be interpreted as exploratory associations. In DFS analyses, HRs greater than 1 indicate a higher hazard of a composite DFS event, defined as recurrence or death. Statistically significant results (*p* < 0.05) are presented in bold.

## Data Availability

The data presented in this study are available on request from the corresponding author. The data are not publicly available due to privacy and ethical restrictions.
